# Supplementary material for: Mental Health Literacy and Positive Mental Health in Adolescents: A Correlational Study
Source: Int J Environ Res Public Health. 2022 Jul 3;19(13):8165. doi: 10.3390/ijerph19138165 (PMC9266633; doi:10.3390/ijerph19138165)
Supplement: Supplementary file 1 [file ijerph-19-08165-s001.zip › Table S1.pdf]

**Table S1.** Sample Characteristics (*n* = 260).

| | *n* | % | Min. | Max. | Mean (SD) |
|---|---|---|---|---|---|
| Age | | | 10 | 19 | 14.07 (1.96) |
| Sex | | | | | |
| Female | 145 | 55.8 | | | |
| Male | 115 | 44.2 | | | |
| Year of School | | | | | |
| 5th | 28 | 10.8 | | | |
| 6th | 7 | 2.7 | | | |
| 7th | 42 | 16.2 | | | |
| 8th | 50 | 19.2 | | | |
| 9th | 62 | 24.2 | | | |
| 10th | 27 | 10.4 | | | |
| 11th | 28 | 10.8 | | | |
| 12th | 15 | 5.8 | | | |
| Employed Father | | | | | |
| Yes | 249 | 95.8 | | | |
| No | 11 | 4.2 | | | |
| Employed Mother | | | | | |
| Yes | 236 | 90.8 | | | |
| No | 24 | 9.2 | | | |
| MH problem | | | | | |
| Yes | 4 | 1.5 | | | |
| No | 256 | 98.5 | | | |
| Recourse to a Health Service due to MH problem (last 3 months) | | | | | |
| Yes | 6 | 2.3 | | | |
| No | 254 | 97.7 | | | |
| Psychologist or psychiatrist monitoring | 79 | 30.4 | | | |
| Yes | 181 | 69.6 | | | |
| No | | | | | |
| Contact with someone with a MH problem | | | | | |
| Yes | 126 | 48.5 | | | |
| No | 134 | 51.5 | | | |
| Hours of sleep/day | | | | | 8.46 (1.19) |
| Taking medication for MH | | | | | |
| Yes | 5 | 1.9 | | | |
| No | 255 | 98.1 | | | |
| Regular exercise | | | | | |
| Yes | 190 | 73.1 | | | |
| No | 70 | 26.9 | | | |
| No. of meals/day | | | | | 4.33 (0.94) |
| Daily fruit/vegetable intake | | | | | |
| Yes | 219 | 84.2 | | | |
| No | 41 | 15.8 | | | |
| Hours online/day | | | | | 5.60 (4.50) |
| Hours in front of a screen/day | | | | | 5.75 (3.63) |
| Victim of violence | | | | | |
| Yes | 47 | 18.1 | | | |
| No | 213 | 81.9 | | | |
| Friends at school | | | | | |
| Yes | 259 | 99.6 | | | |
| No | 1 | 0.4 | | | |

**Table S1.** (cont.)

| | *n* | % | Min. | Max. | Mean (SD) |
|---|---|---|---|---|---|
| Friends out of school | | | | | |
| Yes | 258 | 99.2 | | | |
| No | 2 | 0.8 | | | |
| Consumption of alcoholic drinks | | | | | |
| Yes | 24 | 9.2 | | | |
| No | 236 | 90.8 | | | |
| Tobacco consumption | | | | | |
| Yes | 9 | 3.5 | | | |
| No | 251 | 96.5 | | | |
| MH self-perception | | | 1 | 5 | 4.25 (0.89) |
| PH self-perception | | | 1 | 5 | 4.13 (0.82) |
| Body image self-perception | | | 1 | 5 | 3.73 (0.96) |
| PH self-perception during confinement | | | 1 | 5 | 2.95 (0.87) |
| MH self-perception during confinement | | | 1 | 5 | 3.07 (0.67) |

Abbreviations: Max., maximum; Min., minimum; MH, mental health; *n*, number of cases; No., number; PH, physical health; %, percent; SD, standard deviation.
