# Supplementary material for: Mental Health Literacy and Positive Mental Health in Adolescents: A Correlational Study
Source: Int J Environ Res Public Health. 2022 Jul 3;19(13):8165. doi: 10.3390/ijerph19138165 (PMC9266633; doi:10.3390/ijerph19138165)
Supplement: Supplementary file 1 [file ijerph-19-08165-s001.zip › Table S2.pdf]

Table S2. MHKQ – descriptive statistics (n = 260).

Item	n	%	Min.	Max.	Mean	SD
**Knowledge about the characteristics of mental health and mental disorders**						
1. Mental health is a component of health	260		1	5	4.80	0.67
2. Mental disorders are caused by incorrect thoughts	260		1	5	2.57	1.22
3. Many people have mental problems, but they don't realize it	260		1	5	4.17	0.93
4. All mental disorders are caused by external stressors	260		1	5	3.12	1.16
5. Components of mental health include normal intelligence, stable mood, a positive attitude, quality interpersonal relationships, and adaptability	260		1	5	4.15	1.05
6. Most mental disorders cannot be cured	260		1	5	3.24	1.18
7. Psychological or psychiatric services should be sought if you suspect the presence of mental problems or disorders	260		1	5	4.64	0.77
8. Psychological problems can occur at any age	260		1	5	4.66	0.78
9. Mental disorders and psychological problems cannot be avoided	260		1	5	3.13	1.32
10. In severe mental disorders (eg, schizophrenia), medications should only be taken for a certain period of time	260		1	5	3.43	1.14
11. Positive attitudes, good interpersonal relationships and a healthy lifestyle can help maintain mental health	260		1	5	4.68	0.77
12. Individuals with a family history of mental disorders are at increased risk for psychological and mental disorders	260		1	5	3.70	1.13
13. Psychological problems in adolescents do not influence academic results	260		1	5	4.22	1.15
14. Middle-aged or elderly individuals are unlikely to develop psychological problems and mental disorders	260		1	5	3.89	1.24
15. Individuals with weak temperaments are more likely to have mental problems	260		1	5	3.39	1.08
16. Mental problems or disorders can occur when an individual is under psychological stress or faces a significant situation in their life	260		1	5	4.30	0.96
Total Score			16	80	62.03	6.27
**Awareness of mental health promotion activities**						
17. Have you ever heard about World Mental Health Day?						
Yes	168	64.6				
No	92	35.4				
18. Have you heard about the International Day against Drug Abuse and Illicit Drug Trafficking?						
Yes	98	37.7				
No	162	62.3				
19. Have you ever heard about the International Day for the Prevention of Suicide?						
Yes	125	48.1				
No	135	51.9				
20. Have you ever heard about the World Sleep Day?						
Yes	77	29.6				
No	183	70.4				

Abbreviations: Max., maximum; Min., minimum; n, number of cases; SD, standard deviation; %, percent.
