# Supplementary material for: Mental Health Literacy and Positive Mental Health in Adolescents: A Correlational Study
Source: Int J Environ Res Public Health. 2022 Jul 3;19(13):8165. doi: 10.3390/ijerph19138165 (PMC9266633; doi:10.3390/ijerph19138165)
Supplement: Supplementary file 1 [file ijerph-19-08165-s001.zip › Table S3.pdf]

Table S3. PMHQ – descriptive statistics (n = 260).

Factor/Item	n	Min.	Max.	Mean	SD
**F1. Personal satisfaction**				27.45	4.22
4. I like me the way I am*	260	1	4	3.19	0.91
6. I feel able to explode	260	1	4	3.22	0.90
7. For me life is boring and monotonous	260	1	4	3.49	0.74
12. I see my future with pessimism	260	1	4	3.59	0.78
14. I consider myself a less important person than the other people around me	260	1	4	3.53	0.74
31. I think I'm useless	260	1	4	3.61	0.71
38. I feel dissatisfied with myself	260	1	4	3.43	0.81
39. I feel dissatisfied with my physical appearance	260	1	4	3.38	0.80
**F2. Pro-social attitude**				17.87	2.21
1. It's hard for me to accept others when they have different attitudes from mine	260	1	4	3.48	0.73
3. It's hard for me to listen to people's problems	260	1	4	3.70	0.62
23. I think I'm a trustworthy person*	260	1	4	3.71	0.62
25. I think about the needs of others*	260	1	4	3.27	0.86
37. I like to help others*	260	1	4	3.70	0.63
**F3. Self-control**				14.96	3.20
2. Problems block me easily	260	1	4	3.18	0.75
5. I can control myself when I have negative emotions*	260	1	4	2.77	0.91
21. I am able to control myself when I have negative thoughts*	260	1	4	2.99	0.86
22. I am able to maintain good self-control in conflict situations that arise in my life*	260	1	4	3.00	0.84
26. In the presence of unfavourable pressures from outside I am able to maintain my personal balance*	260	1	4	3.02	0.84
**F4. Autonomy**				15.69	3.01
10. I'm very concerned about what people think of me	260	1	4	3.04	0.90
13. The opinions of others influence me a lot when making my decisions	260	1	4	3.19	0.80
19. I worry that people criticize me	260	1	4	3.06	0.92
33. I have difficulties in having personal opinions	260	1	4	3.53	0.80
34. When I have to make important decisions, I feel very insecure	260	1	4	2.87	0.91
**F5. Problem-solving and self-actualization**				29.32	4.46
15. I am able to make the decisions for myself*	260	1	4	3.36	0.83
16. I try to remove the positive aspects of the "bad" things that happen to me*	260	1	4	2.87	0.98
17. I try to improve myself*	260	1	4	3.57	0.77
27. When there are changes in my life, I try to adapt*	260	1	4	3.42	0.72
28. Faced with a problem, I am able to request information*	260	1	4	3.16	0.83
29. The changes that usually occur in my daily life stimulate me*	260	1	4	2.75	0.90
32. I try to develop and enhance my good attitudes*	260	1	4	3.55	0.68
35. I can say no when I want to say it*	260	1	4	3.21	0.87
36. When I have a problem, I try to find possible solutions*	260	1	4	3.45	0.74
**F6. Interpersonal relationship skills**				22.97	3.58
8. It's hard for me to give emotional support	260	1	4	3.50	0.79
9. I have difficulties establishing satisfactory interpersonal relationships with some people	260	1	4	3.52	0.69
11. I believe that I have a lot of capacity to put myself in the shoes of others and understand their responses*	260	1	4	2.95	0.94
18. I consider myself a good advisor*	260	1	4	3.13	0.86
20. I consider myself a sociable person*	260	1	4	3.20	0.92
24. It's hard for me to understand the feelings of others	260	1	4	3.43	0.77
30. I have difficulties in relating openly with my teachers/boss	260	1	4	3.23	0.95

Abbreviations: Max., maximum; Min., minimum; n, number of cases; SD, standard deviation; *, inverted items.
